# Electrospun Weak Anion-exchange Fibrous Membranes for Protein Purification

**DOI:** 10.3390/membranes10030039

**Published:** 2020-03-01

**Authors:** Shu-Ting Chen, S. Ranil Wickramasinghe, Xianghong Qian

**Affiliations:** 1 Department of Chemical Engineering, University of Arkansas, Fayetteville, AR 72701, USA; sc068@email.uark.edu; 2 Department of Biomedical Engineering, University of Arkansas, Fayetteville, AR 72701, USA; swickram@uark.edu

**Keywords:** anion exchange, electrospun membrane, UV-initiated polymerization, protein purification

## Abstract

Membrane based ion-exchange (IEX) and hydrophobic interaction chromatography (HIC) for protein purification is often used to remove impurities and aggregates operated under the flow-through mode. IEX and HIC are also limited by capacity and recovery when operated under bind-and-elute mode for the fractionation of proteins. Electrospun nanofibrous membrane is characterized by its high surface area to volume ratio and high permeability. Here tertiary amine ligands are grafted onto the electrospun polysulfone (PSf) and polyacrylonitrile (PAN) membrane substrates using UV-initiated polymerization. Static and dynamic binding capacities for model protein bovine serum albumin (BSA) were determined under appropriate bind and elute buffer conditions. Static and dynamic binding capacities in the order of ~100 mg/mL were obtained for the functionalized electrospun PAN membranes whereas these values reached ~200 mg/mL for the functionalized electrospun PSf membranes. Protein recovery of over 96% was obtained for PAN-based membranes. However, it is only 56% for PSf-based membranes. Our work indicates that surface modification of electrospun membranes by grafting polymeric ligands can enhance protein adsorption due to increased surface area-to-volume ratio.

## 1. Introduction

The global market of biologics, particularly, protein therapeutics is growing rapidly [1,2]. The production of protein-based human therapeutics such as monoclonal antibodies (mAbs) and Fc-fusion proteins involves cultivating mammalian cells such as Chinese hamster ovary cells (CHO) in complex cell culture suspension [3]. The desired protein therapeutics are typically secreted by the cells into the suspension media. The protein product must then be recovered and purified. The rapid advancement in upstream cell culture operations has led to a significant increase in product titers. However, this high-level of productivity is accompanied by establishing much higher cell density [4,5] which places a much larger burden on the traditional downstream clarification and purification operations. Downstream processing becomes the bottleneck in the production of protein therapeutics and contributes significantly to the production cost [6]. High capacity and high recovery downstream purification unit operations are essential for the cost-effective purification of biologics.

Ion-exchange (IEX) and hydrophobic interaction (HIC) chromatography are routinely used during the downstream purification of protein therapeutics. After the initial capturing step using protein A chromatography, the feed stream typically is further processed by additional polishing steps such as IEX and HIC chromatography operated under flow-through mode to further reduce the host cell proteins (HCPs), DNA, aggregates and other impurities present [7]. However, resin-based packed-bed chromatography suffers from high pressure drop and slow pore diffusion which leads to longer processing time and potentially denaturation of the product. Membrane adsorbers are promising technologies to replace resin-based chromatography [8,9,10,11,12,13,14]. Membrane-based IEX and HIC adsorbers can overcome afore-mentioned limitations [15,16]. Moreover, the performance of membrane adsorbers is largely independent from the feed flow rate. However, membrane capacity is typically lower compared to that of resin. Significant efforts have been dedicated to develop high binding capacity and/or high recovery membrane adsorbers by grafting ligands on membrane substrates using UV-initiated polymerization or atom-transfer radical polymerization (ATRP) [8,9,10,11,12,13,14,17,18,19].

Electrospun membranes provide a 3-D scaffold which enhances surface area to volume ratio for protein adsorption. Electrospinning has attracted attention as a versatile and robust method for fabricating nanofibrous membranes [20,21,22]. Compared with membranes produced through temperature or non-solvent induced phase inversion processes [23], electrospun nanofibrous membranes have a much higher porosity due to their unique interconnected fiber structures. Using the electrospun membrane as a substrate to attach ligands can increase the available grafting area for protein binding. Previous study [24] evaluated the performance of electrospun polyethersulfone (PES) affinity membranes. These PES affinity membranes demonstrated a high specific binding selectivity for IgG molecules and low non-specific protein adsorption as well as low flow-through pressure drop due to their large pore sizes. Another study [25] reported the fabrication of electrospun carbon nanofibrous mats, a promising alternative to the packed-bed media for bioseparation applications. The binding capacity for lysozyme of the mats reached over 200 mg/g of adsorption media. In addition, these mats showed high feed flow rate and low pressure drop due to their large pore sizes. Earlier work [26] also tested the effects of compression and the number of bed layers for the dynamic binding capacity of regenerated cellulose based IEX electrospun membranes. The highest dynamic binding capacity for lysozyme reached ~21 mg/mL for carboxylate adsorbents at a compressive pressure of 1 MPa. An increase in the compressive pressure decreased dynamic binding capacity. Moreover, a decrease in dynamic binding capacity was also observed as the number of bed layers increased from 4 to 12 due to the reduction in surface area resulting from membrane compression. High static binding capacity reaching 284 mg/g of adsorbents for lysozyme obtained from grafting critic acid on electrospun ethylene-vinyl alcohol (EVOH) nanofibrous membranes [27]. Previous work focused largely on grafting monomeric cation-exchange ligands on electrospun membrane substrates. No systematic investigation has been conducted on the performance of grafted polymeric anion-exchange electrospun membranes. Here the effects of ligand chain length, chain density as well as the substrate material and fiber diameter on protein binding capacity are studied.

There are several approaches to graft ligands on electrospun membrane substrates. Previous studies [24,25,26,27] conjugated a monomeric acid, base or affinity ligand directly to the functional group on the substrate. However, there has been significant work [8,9,10,11,12,13,14,17,18,19] on surface modification by grafting polymers on the flat sheet membrane substrates using ATRP and UV-initiated polymerizations. These results show that there exists an optimal ligand chain density and chain length for maximizing protein binding capacities for IEX, affinity and HIC membrane chromatography [19]. Surface modification with UV initiated reaction or polymerization is fast and robust with extensive application for IEX membranes [28,29]. Previous review [16] showed that the UV irradiation time, initiator concentration and the overall grafting degree affect protein binding capacity and membrane throughput significantly. Too high a grafting density can result in low binding capacity due to ligand aggregation and reduced protein accessibility. At relatively low grafting degree, ~100 mg/mL protein binding capacity of BSA can be obtained. Besides ligand, substrate morphology and porosity also play an important role [11]. Previous work [30] investigated the effects of degree of glycidyl methacrylate (GMA) grafting of non-woven substrate on the kinetics of non-specific protein adsorption. Their results indicate that higher surface area nonwoven substrate promotes faster protein adsorption.

As earlier work [24,25,26,27] focused on grafting monomeric cation exchange ligands, our work of grafting and optimizing polymeric anion exchange ligands on the electrospun membrane substrates is novel even though established surface chemistry has been adopted. Since ATRP requires inert condition and is difficult to implement at a large scale, UV-initiated polymerization on electrospun membranes to form IEX membranes was investigated here. In particular, the weak anion-exchange membranes were fabricated by grafting poly(glycidyl methacrylate) to the electrospun membrane substrates via UV-initiated polymerization followed by the addition of diethylamine (DEA) via a ring-opening reaction. Both polyacrylonitrile (PAN) and polysulfone (PSf) substrates were investigated. The properties of functionalized membranes were characterized using Fourier transform infrared spectroscopy (FTIR), scanning electron microscopy (SEM) and zeta potential measurement. Furthermore, adsorption experiments were performed to determine the static and dynamic protein binding capacities of the functionalized membranes.

## 2. Materials and Method

### 2.1. Materials

Polysulfone (PSf, MW 60 kg/mol) in powder form as membrane material was purchased from BASF (Ludwigshafen, Germany). Polyacrylonitrile (PAN) in powder form as membrane material was kindly provided by R&D membrane center from Chung Yuan Christian university, Taiwan [31]. N, N-dimethylformamide (DMF), methanol (ACS grade), Tris (hydroxymethyl) aminomethane (Tris, biotechnology grade), and sulfuric acid (ACS grade) were purchased from VWR (Radnor, PA, USA). glycidyl methacrylate (GMA), sodium phosphate dibasic (Reagent Plus, ≥99.0%), sodium phosphate monobasic monohydrate and benzophenone (BP) were purchased from Sigma-Aldrich (St. Louis, MO, USA). Inhibitors in GMA were removed through a pre-packed inhibitor remover column (Sigma-Aldrich, St. Louis, MO, USA) prior to use. 1-Butanol (>99% ACS grade) and diethylamine (DEA, reagent grade 99%) were purchased from Alfa Aesar (Ward Hill, MA, USA). Bovine serum albumin (BSA) were acquired from Lee BioSolution (Maryland Heights, MO, USA). All chemicals were used without further purifications except GMA. Deionized water (DI) was obtained from Milli-Q ultrapure water purification system (Millipore, Billerica, MA, USA).

### 2.2. Methods

#### 2.2.1. Fabrication of Fibrous Membranes

Electrospinning was used to fabricate polysulfone (PSf) nanofibrous membranes. PSf powders were dissolved in DMF (20 w/v%) under continuous stirring at 70 °C for about 24 h until a homogeneous solution was obtained following our earlier work [32]. The mixed solution was subsequently used for the fabrication of electrospun membranes using a syringe pump. Here the pump flow rate, applied voltage, needle gauge, and the distance between the needle tip and collector were fixed at 0.5 mL h^−1^, 25 kV, 22-gauge needle, and 25 cm, respectively for the fabrication of PSf membranes. The reaction temperature was maintained between 21–25 °C and the relative humidity at 23% during the electrospinning of PSf. The nanofiber mat was prepared by collecting nanofibers over a period of 4 h.

To fabricate PAN nanofibrous membranes, PAN powder was dissolved in DMF (8 w/v%) with stirring to form a homogenous solution. The casting solution was then placed into a syringe pump and electrospun onto the aluminum foil following the similar procedure to the fabrication of PSf membrane. The flow rate, applied voltage and the distance between the needle tip and the collector are 0.6 mL h^−1^, 20 kV and 15–20 cm respectively. The nanofiber mat was prepared by collecting nanofibers for 4 h.

#### 2.2.2. UV-Initiated Polymerization of GMA

**Scheme 1:**
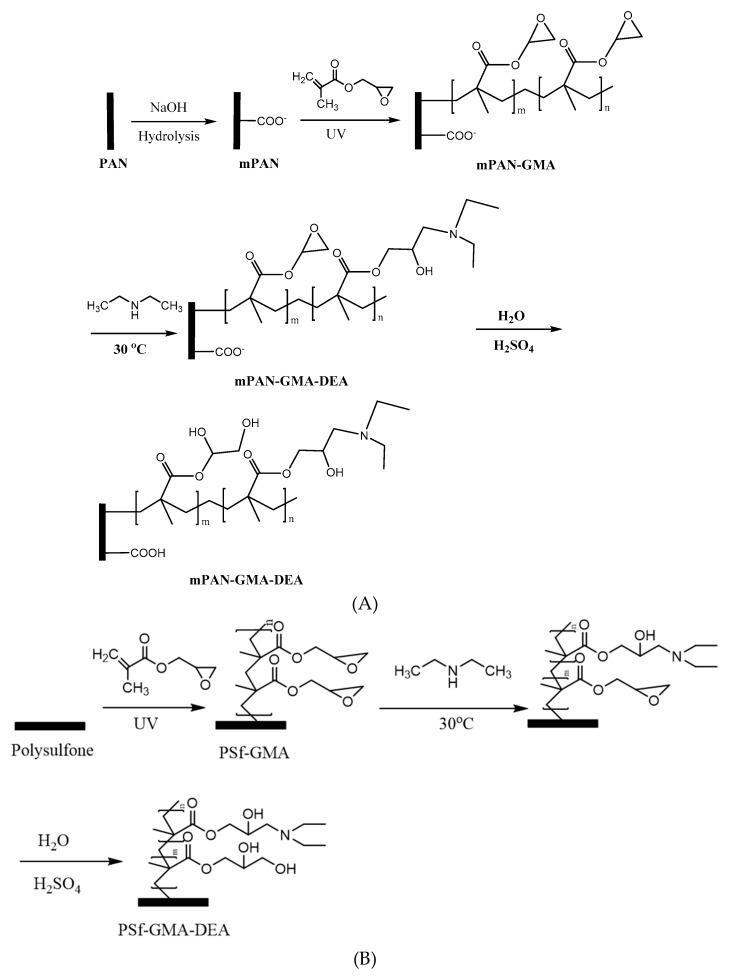
Surface modification of hydrolyzed electrospun polyacrylonitrile (PAN) membrane (**A**); and electrospun polysulfone (PSf) membrane (**B**).

The PSf and PAN nanofibrous membranes were further surface modified with tertiary amine groups. Two step modification procedure is illustrated below in Scheme 1 following the previous chemistry [33]. The PSf fibrous and PAN fibrous membranes were first washed with methanol and dried at 40 °C under reduced pressure using a vacuum oven. PAN membranes were hydrolyzed prior to the use in 2 M NaOH solution at 50 °C for 2 h. The hydrolyzed membranes were then rinsed with DI water until the pH of the water reached 7.0. The membranes were subsequently dried in a vacuum oven before modification. Butanol was used as a solvent for UV initiated grafting of GMA with benzophenone (BP) as the UV initiator. A grafting solution contained 20% (v/v) GMA and 1% (w/v) BP. A pre-weighed membrane (6.2 cm^2^, ~13 mg) was dispersed evenly with GMA grafting solution until it is completely soaked. The membrane was then sandwiched between two petri dishes. One side of the membrane was exposed to UV lamp (model UVAPRINT 100, Honle UV technology, Marlboro, MA, USA) with intensity of 44.5 mW/cm^2^. The distance between the membrane and the lamp was maintained to be around 40 cm. After completion of the predetermined reaction time, the membrane was washed in methanol to remove any unreacted GMA and BP. Thereafter, the membrane was finally dried in a vacuum oven for 4 h at 40 °C. Thereafter, the membrane was grafted with GMA on the other side of the membrane following the same procedure.

GMA-grafted membranes (PSf-GMA and PAN-GMA) were then immersed in a 50% (v/v) diethylamine (DEA) aqueous solution at 30 °C with gentle shaking overnight. Thereafter the membranes were washed repeatedly with a DI water and methanol mixture to remove any unreacted species. To reduce nonspecific protein binding, these modified membranes were then hydrolyzed in a 0.1 M sulfuric acid solution at 50 °C overnight to convert any remaining epoxy group to hydroxyl groups. The membranes were again washed with DI water after hydrolysis [33]. After that, the membranes were dried at 40 °C under reduced pressure using vacuum oven. The grafting degree was calculated based on the amount of GMA grafted as shown in Equation (1) below where W_0_ is the original membrane weight (mg) and W_1_ is the weight after GMA and DEA grafting (mg). Triplicate measurements were conducted to obtain the accuracy of the standard deviation.

(1) $$\mathrm{Degree}\;\mathrm{of}\;\mathrm{grafting}\;\left(\%\right)=\frac{W_{1}-W_{0}}{W_{0}}$$

Similarly, nanofibrous PAN membranes were modified. However, PAN membranes were hydrolyzed before use in 2 M NaOH solution at 50 °C for 2 h. The hydrolyzed membranes were then rinsed in DI water until the pH of the water reached 7.0. The membranes were subsequently dried in a vacuum oven before modification with GMA and DEA.

#### 2.2.3. Membrane Morphology

Surface and cross-sectional morphology of these fabricated nanofibrous membranes were imaged using a scanning electron microscopy (SEM). Cross-sectional images of the membranes were obtained by fracturing the membranes in liquid nitrogen.

#### 2.2.4. Zeta Potential

The surface charge in terms of Zeta potential on the membrane was measured using Beckman Coulter Delsa NanoHC (Brea, CA, USA). A flat cell was used for measuring the zeta potential on the membrane surface. Dry membranes were immersed in the buffer solution with predefined pH value. NaCl solution was used for calibration and triplicate measurements were carried out for each membrane [34].

### 2.3. Membrane Binding Capacity Determination

#### 2.3.1. Static Protein Binding Capacity Measurement

The static binding capacity were measured for these modified electrospun membranes using bovine serum albumin (BSA) as a model protein. BSA has an isoelectric point (pI) of 4.7 and is negatively charged at neutral pH. Static protein adsorption isotherms were measured on unmodified and GMA-DEA modified membranes. Six different initial BSA concentrations at 0.5, 1.0, 1.5, 2.0, 2.5 and 3.0 mg/mL in 20 mM Tris-HCl buffer at pH 7.0 were used to measure the amount of protein adsorbed by the membranes. The membranes were cut into 4.9 cm^2^ disks. Each membrane was placed in a 60 mL glass bottle from VWR (Radnor, PA, USA). All membranes were first equilibrated with a pH 7 tris buffer for 1 h. Thereafter, all membranes were challenged with a specified protein solution and equilibrated for 20 h at room temperature under gentle shaking. The equilibrium concentrations of BSA solutions were first measured using UV absorbance at 280 nm with a UV–VIS spectrophotometer (Thermo Scientific™ GENESYS 10S UV-Vis, Waltham, MA, USA). BSA binding capacity of the membrane at each protein concentration was calculated below:

(2) $$\mathrm{Binding}\;\mathrm{capacity}=\frac{\mathrm{Amount}\;\mathrm{of}\;\mathrm{protein}\;\mathrm{bound}\;\mathrm{to}\;a\;\mathrm{membrane}}{\;\mathrm{membrane}\;\mathrm{volume}\;}$$

#### 2.3.2. Dynamic Binding Capacity Measurement

Protein stock solutions were prepared by dissolving 30 mg of BSA protein in 10 mL of 20 mM Tris-HCl buffer (pH 7.0, Buffer A), which contained no other salt. Then this stock solution of BSA was added to the 20 mM Tris-HCl buffer containing 1 M of NaCl (Buffer B) to obtain a solution of 3 mg/mL protein solution. All the buffers and protein solutions were then filtered with Whatman 0.2 μm PES membrane before the dynamic protein binding test. Membranes were soaked in buffer A for 10 min before use. Each run used four membranes packed in a stainless-steel flow cell (Mustang Coin ^®^ module, Pall Corporation, New York, NY, USA). A flow distributor was placed on each side of the membrane stack to obtain a uniform flow across the membranes. All runs were conducted with ÄKTA FPLC (GE Healthcare Bio-Sciences Corp, Chicago, IL, USA). The runs were automated using the Unicorn software 7.3 for binding and elution. The system was initially equilibrated with buffer A (adsorption buffer) at a flow rate of 1 mL/min for 10 min. Feed protein solution containing 3 mg/mL BSA was then loaded onto the membrane at a flow rate of 1 mL/min for 10 min for a total of 10 mL feed. Unbound proteins were then washed away from the membrane using buffer A (adsorption buffer) for 10 min at 1 mL/min, followed by elution with buffer B (elution buffer) at a flow rate of 1 mL/min for 10 min. The runs ended when the UV absorbance at 280 nm reached a constant. Three fractions were collected including loading, washing as well as elution. Protein concentrations in these fractions were determined by UV at 280 nm. Protein binding capacity was calculated using Equation (2) whereas protein recovery was determined using the following formula:

(3) $$\mathrm{Recovery}=\frac{\mathrm{Amount}\;\mathrm{of}\;\mathrm{eluted}\;\mathrm{protein}\;}{\;\mathrm{Amount}\;\mathrm{of}\;\mathrm{protein}\;\mathrm{bound}\;\mathrm{to}\;\mathrm{the}\;\mathrm{membrane}}$$

## 3. Results and Discussion

### 3.1. Membrane Characterization 

Chemical composition, surface morphology and membrane properties of the PSf and PAN based electrospun nanofibrous membranes were characterized using FTIR, scanning electron microscopy (SEM) and zeta potential measurements.

#### 3.1.1. Chemical Composition of Membrane Surface

**Figure 1:**
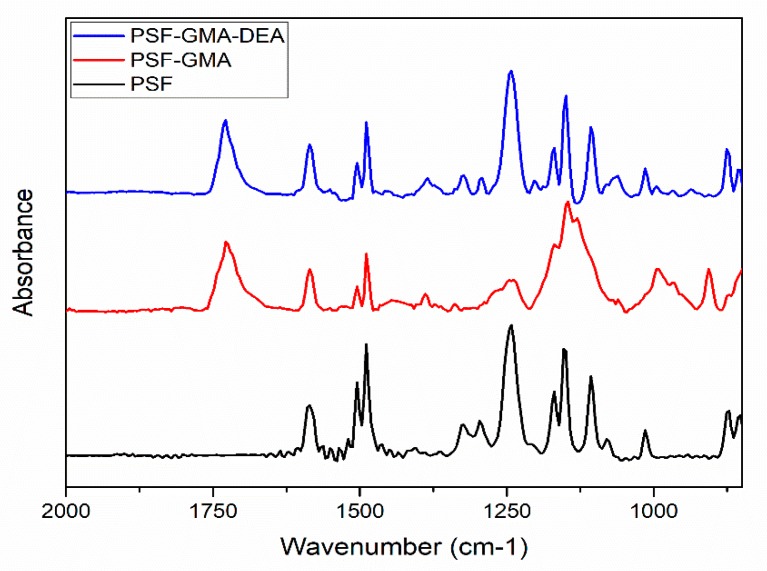
FTIR spectra of PSf base membrane (black), after modification with glycidyl methacrylate (GMA) (red), and further modification with diethylamine (DEA) (blue) to form PSf-GMA-DEA membrane.

FTIR spectra for the fabricated nanofibrous PSf membranes are shown in Figure 1. The FTIR spectrum of the base PSf membrane (black line, bottom) exhibits a number of characteristic peaks. After grafting GMA on the membrane (red line, middle), two distinctive new peaks can be observed at 905 cm^−1^ and 1730 cm^−1^ respectively [35]. The 905 cm^−1^ peak comes from the epoxy group whereas the 1730 cm^−1^ peak arises from the ester carbonyl group after modification with GMA [36]. At the same time, the 1235 cm^−1^ peak from the tertiary amine from the base membrane becomes less prominent. The FTIR spectra indicate that GMA was successfully grafted onto the PSf membrane. After further modification with DEA followed by converting the remaining epoxy to OH group, a prominent tertiary amine peak at 1235 cm^−1^ was again observed. The epoxy peak at 905 cm^−1^ almost disappears indicating successful conversion and modification to form PSf-GMA-DEA membranes [37].

**Figure 2:**
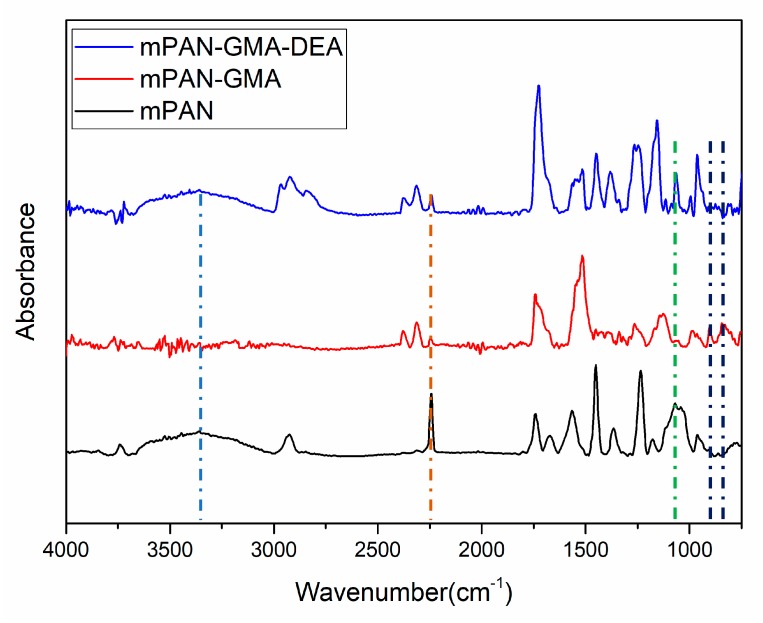
FTIR spectra of hydrolyzed PAN membrane (black), PAN membrane modified with GMA (red) and membrane modified with GMA-DEA (blue).

FTIR spectra of PAN membranes before and after modification are shown in Figure 2. The base PAN membrane (black) before hydrolysis and PAN-GMA-DEA membrane (blue) all exhibit a broad peak at around 3400 cm^−1^ due to the presence of surface –OH groups. After GMA modification (red), the broad –OH peak almost disappears. The carbonyl group from the ester group at 1730 cm^−1^ becomes significantly enhanced after the modification. The sharp peak at 2243 cm^−1^ arises from the stretching of the residue –CN group in the base membrane (black). This peak becomes much weaker after the membrane surface is modified with GMA (red). The broad peak in the range of 1065 cm^−1^ was attributed to the stretching of C–N bonds [33]. The bending peaks from the epoxy group after GMA modification were observed in the range of 904 and 847 cm^−1^ (red) [38]. The amine peak at 1230 cm^−1^ becomes much weaker upon surface modification with GMA, but with much enhanced intensity after the DEA modification. The FTIR spectra again suggest successful modification of the PAN membrane with GMA and DEA.

#### 3.1.2. Membrane Structure and Morphology

**Figure 3:**
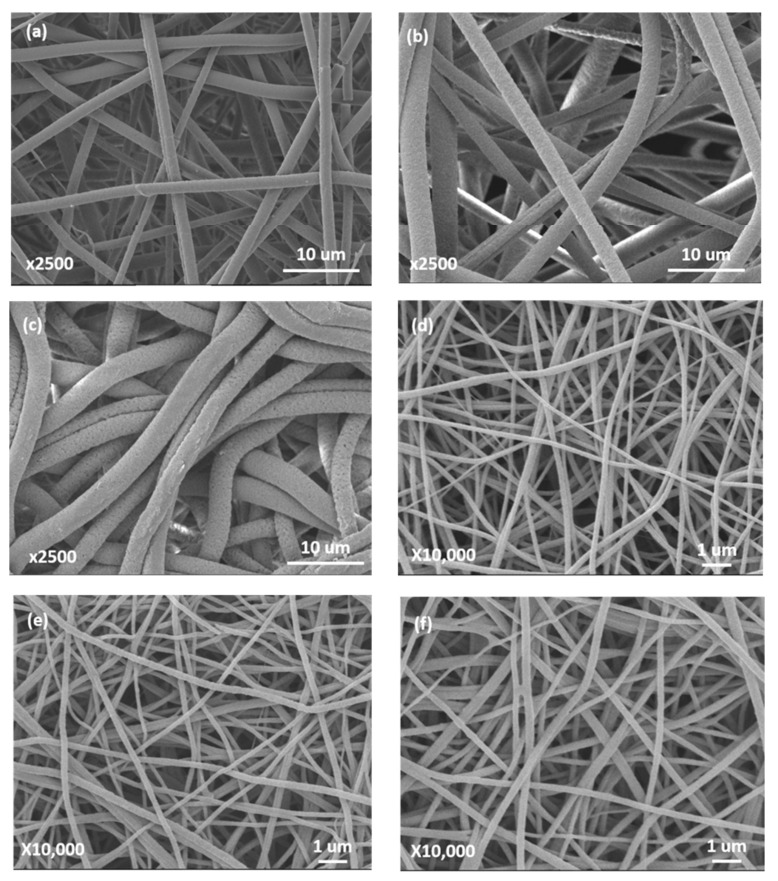
SEM morphology of unmodified PSf (**a**), PSf-GMA with 7 min UV (**b**), PSF-GMA-DEA (**c**), unmodified mPAN (**d**), mPAN-GMA with 15 min UV (**e**), and mPAN-GMA-DEA (**f**).

Scanning electron microscopy (SEM) images of electrospun nanofibrous PSf and PAN membranes before and after surface modifications were shown in Figure 3. It can be seen that PSf based membranes exhibited uniform nanofibrous structures. For the base PSf membrane, the average diameter of PSf fibers is in the range of 1.25 μm ± 0.23 μm. However, the fiber diameter increased significantly as more modifications were performed on the base membrane as can be seen from Figure 3a–c. The fiber diameter for PSf-GMA-DEA membrane is almost twice as much as that of the base PSf membrane. Moreover, the fiber surface also becomes visibly rougher after GMA and DEA modification. This dramatic increase in fiber diameter can be explained by the polymerization process adopted here. When grafting the polymer, the UV initiator BP and monomer GMA were mixed together in the prepared solution, in which the membranes were soaked to uptake the mixtures. In addition to grafting GMA polymers chemically bonded to the membrane substrate, this UV-initiated polymerization can also lead to the polymerization of the free GMA polymers adsorbed physically on the surface of the membrane substrate [39]. The physically adsorbed free GMA polymers lead to the significant increase in fiber diameter and increase roughness since the polymer chains were not tightly packed. A two-step process involving the initial initiator adsorption followed by the subsequent polymerization can reduce the probability of forming free polymer chains.

The mPAN electrospun membranes exhibit clearly a thinner fibrous structure than PSf membranes as shown in Figure 3d–f. The average diameter of mPAN fibers was estimated to be in the range of 150 to 340 nm before surface modification. Upon 15 min of UV initiated polymerization of grafting GMA, average fiber diameter only increased slightly. The significantly reduced grafting degree and the less hydrophobic nature of the mPAN membrane compared to that of PSf membrane likely lead to the much smaller fiber diameter. Further modification with DEA, fiber size has visibly increased. This is probably due to fact that reaction of DEA to the epoxy group resides in each GMA monomer unit leading to a substantial increase in the grafted polymer molecular weight.

#### 3.1.3. Zeta Potential

**Figure 4:**
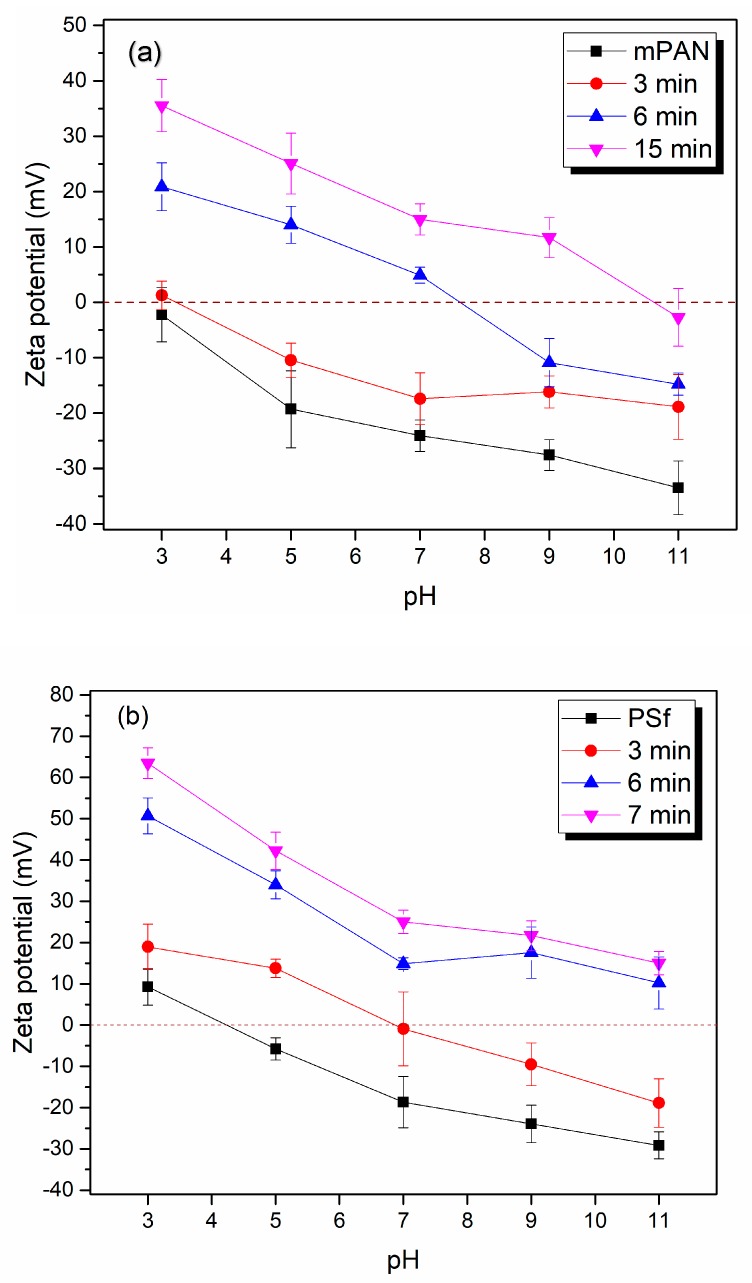
(**a**) Zeta potential of mPAN base membrane, modified mPAN-GMA-DEA membranes with 3, 6, and 15 min UV polymerization, (**b**) Zeta potential of PSf base membrane, modified PSf-GMA-DEA membranes with 3, 6, and 7 min UV polymerization.

Zeta potential of the electrospun PAN base membrane and modified membranes at 3, 6, and 15 min UV polymerization times were measured and shown in Figure 4. It can be seen that the base membrane (black) at the pH range between 3 and 11 has a negative surface charge. This is due to the fact that after NaOH hydrolysis, some of the –CN groups on PAN membrane are converted to the negatively charged –COO^−^ groups. After the GMA-DEA surface modification, the zeta potential increases at each pH value increases. This is due to the incorporation of the tertiary amines in the membrane surface leading to an increase in the surface charge. Moreover, the longer the UV polymerization or the GMA chain is, the higher the DEA number incorporated into the surface layer, thereby the higher the number of tertiary amines resides on membrane surface, which in turn leads to a higher zeta potential. After 15 min of UV polymerization and the subsequent DEA addition, the zeta potential becomes largely positive except at pH 11 indicating higher density of the positive charges on the membrane surface and subsequently higher anion-exchange capacity. The zeta potential trend of modified PSf membranes as a function of pH is similar to that of PAN electrospun membranes. However, the former is slightly higher overall than the latter at the same pH value and the same grafting condition.

#### 3.1.4. Grafting Degree

**Figure 5:**
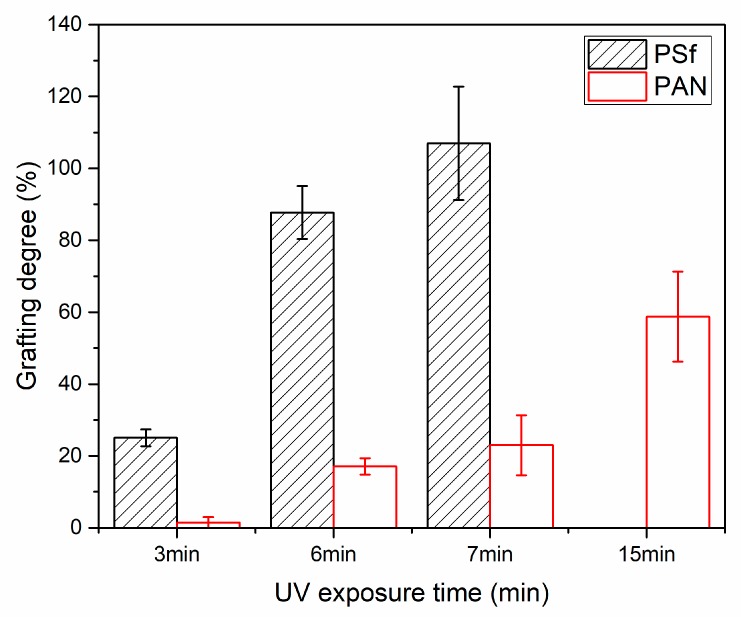
Grafting degree as a function of UV polymerization time for both electrospun PSf and PAN membranes.

Grafting degrees of modified PSf and PAN nanofibrous membranes with respect to UV polymerization times are shown in Figure 5. As mentioned previously, the UV polymerization was conducted on both sides of the membrane in order to functionalize more tertiary amines on the membrane surface. PSf electrospun membrane was found to sustain damages when the UV exposure was longer than 7 min. This is due to the fact that membrane base materials are sensitive to UV light [40]. Longer UV exposure leads to a decreased mechanical strength and reduced chemical stability. As a result, UV polymerization of grafting GMA was conducted for 1, 3, and 7 min only for the PSf membranes. The grafting degree was determined after both GMA and DEA modifications. It can be seen from Figure 5 that when the UV polymerization time increased from 1 to 7 min, the grafting degree increased dramatically from about 25% to over 100% indicating the efficiency of the polymerization. However, as mentioned previously, it is likely that polymers that are not chemically bonded to the membrane surface are formed as well during the polymerization reaction since the initiator and monomer were mixed together.

On the other hand, for PAN electrospun membranes, grafting degree also increases with the increase of UV polymerization time. After 15 min of UV polymerization and subsequent DEA addition, ~55% grafting degree was achieved, significantly less than those grafted on PSf membranes. This difference probably lies in the fact that UV initiated polymerization can occur even without the initiator BP for the PSf membrane. The contact angle for unmodified electrospun PSf membrane was estimated to be 128.0 ± 4.8° whereas it was measured to be 73.1 ± 3.5° for unmodified hydrolyzed PAN membranes. Since multiple factors including surface roughness contribute to water contact angle, contact angles for flat sheet membranes were also measured. Contact angles for flat sheet PSf and PAN membranes are measured to be 70.0 ± 1.7° and 45.0 ± 4.6°, respectively. These observations confirm that the PSf base membrane is more hydrophobic than the PAN membrane in agreement with previous studies [41,42]. As a result, more initiator BP and monomer GMA molecules can be adsorbed on the membrane surface leading to an increased polymer chain density thereby increased grafting degree. Moreover, increased initiator and monomer uptake by the PSf membrane leads to the increased number of free UV-initiator polymer chains.

### 3.2. Static and Dynamic Protein Binding Capacity Tests

**Figure 6:**
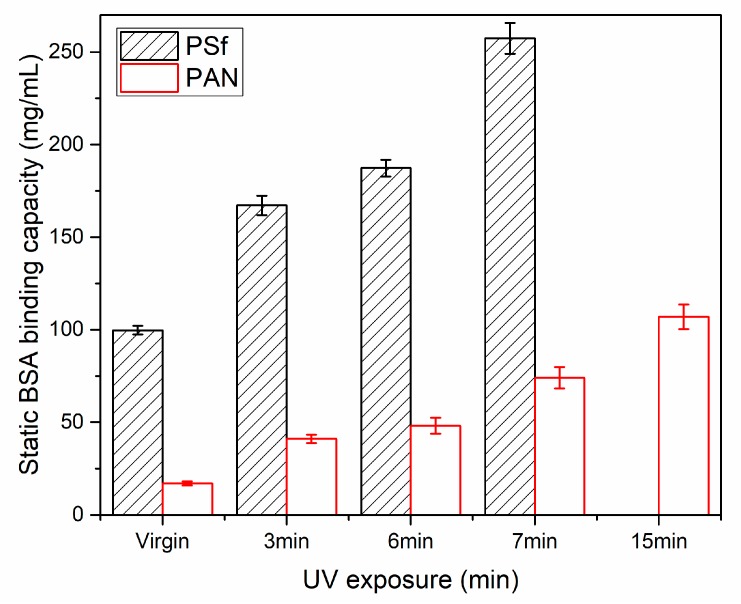
Static binding capacities of unmodified (virgin) and modified PSf and PAN membranes at different UV polymerization times. Error bars represent the standard deviation from 3 measurements. For PAN, the virgin membrane is after NaOH hydrolysis.

Static protein binding tests were conducted for the electrospun base and modified PSf and PAN membranes as a function UV polymerization time as shown in Figure 6. The membranes were equilibrated with the protein solutions for 20 h. The time dependent binding capacities of the two membranes are shown in Figure S1 in the supplementary document. The BSA binding capacity increases as a function of time and reaches a stable value after about 12 h. The unmodified PSf electrospun membrane exhibits non-specific protein binding capacity of close to 100 mg/mL whereas the PAN membrane only has about 20 mg/mL. As indicated previously, PSf base membrane is more hydrophobic than the hydrolyzed electrospun PAN base membrane since PAN possesses negatively charged -COO^–^ groups. After 3, 6 and 7 min of UV initiated polymerization and the final functionalization of DEA, the BSA binding capacity of PSf membrane increased dramatically to about ~260 mg/mL. This significant increase in protein binding capacity is correlated with the more than 100% of the grafting degree observed for the PSf membranes. The overall increase in the protein binding capacity is correlated with the increase in grafting degree. Even though PAN membranes exhibit relatively low protein binding capacity, the overall trend in its binding capacity with regard to grafting degree is still valid. The static binding capacity for modified electrospun PAN membranes increased from ~40 mg/mL after 3 min UV polymerization to about 100 mg/mL after 15 min UV polymerization and the addition of DEA. The high protein binding capacity of PSf-GMA-DEA membrane partly comes from its more hydrophobic base membrane and partly from its higher grafting degree.

The high protein binding capacities of the electrospun and surface grafted PSf-GMA-DEA and PAN-GMA-DEA membranes come partly from their high surface-volume ratio compared to flat sheet membranes. In addition, surface modification which increased the density of tertiary amine is also partly responsible for the enhanced protein binding. This indicates that electrospun nanofibrous membranes with appropriate functionalization are promising for improved performance during downstream protein purification. The high non-specific binding of proteins on PSf-GMA-DEA membrane remains to be a challenge for industrial applications. Moreover, additional investigation is necessary for the separation of protein mixtures with different pIs at appropriate feed conditions.

**Figure 7:**
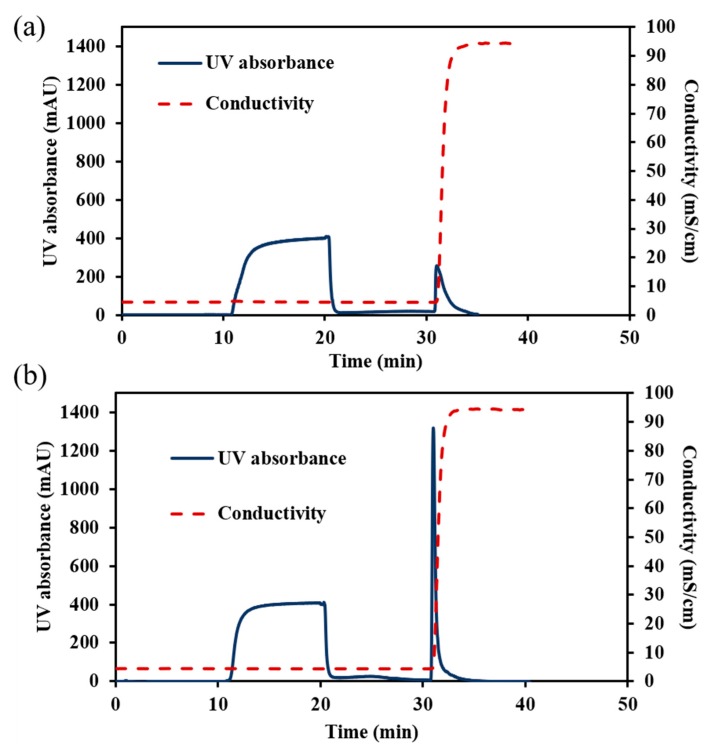
Chromatograms of dynamic protein binding runs using PSf-GMA-DEA with 7 min UV polymerization (**a**), and mPAN-GMA-DEA with 15 min UV polymerization (**b**).

Dynamic binding capacities of the fabricated membranes were determined using FPLC Chromatograms for FPLC runs with both surface-modified electrospun membranes are shown in Figure 7. The PSf-GMA-DEA membrane was surface modified with 7 min of UV polymerization whereas the PAN-GMA-DEA membrane was modified with 15 min of UV polymerization. The PSf-GMA-DEA membrane exhibited a BSA protein binding capacity of 201.3 mg/mL membrane volume as compared to the value of 87.2 mg/mL for the corresponding PAN-GMA-DEA membrane. However, in the case of PSf-GMA-DEA membrane, only ~56% of the BSA was recovered upon elution which was significantly lower than the value of ~96% for the corresponding PAN-GMA-DEA membrane. The protein binding and recovery results are tabulated in Table 1. The fact that PSf-GMA-DEA has a much higher protein binding capacity and relatively lower protein recovery and that PAN-GMA-DEA has a lower protein binding capacity and a much higher recovery agrees with the properties of the membranes and our previous static protein binding tests. The former is more hydrophobic and thus more non-specific protein binding leading to a higher binding capacity but lower recovery. The latter is more hydrophilic with much less non-specific protein binding leading to a lower binding capacity but increased recovery.

## 4. Conclusions

Here PSf and PAN surface modified weak anion-exchange electrospun nanofibrous membranes were fabricated by UV-initiated polymerization. The physiochemical properties of the surface modified membranes were characterized using SEM and FTIR. The changes in surface charge and charge density as a function of UV polymerization time were measured by zeta potential and correlated with the polymer grafting degree. Due to the high substrate porosity resulting from the high surface-to-volume ratio of the nanofibrous materials, the surface modified PSf-GMA-DEA and mPAN-GMA-DEA membranes exhibit high static and dynamics protein binding capacities. BSA protein binding capacity increases as the grafting degree increases in agreement with surface charge measurements. However, after 15 min of UV exposure, the mechanical integrity of PSf membrane is compromised. The PSf-GMA-DEA membrane is more hydrophobic than the mPAN-GMA-DEA membrane leading to its increased non-specific binding of the protein. The BSA recovery of mPAN-GMA-DEA membrane reached over 96% whereas it is only ~56% for the PSf-GMA-DEA. Our study indicates that electrospun nanofibrous mPAN-GMA-DEA membrane is promising as a high capacity high recovery ion-exchange membrane for protein purifications.

## Figures and Tables

**Table 1:** Productivity comparison for different substrate membranes. Error bars represent the standard deviation of calculated values using data from 2 measurements.

Substrate	Dynamic Binding Capacity (BSA, mg/mL Membrane Volume)	BSA Yield (%)
PSf	201.3 ± 4.8	55.9 ± 5.1
PAN	87.2 ± 2.6	96.4 ± 3.2

## Supplementary Materials

The following are available online at https://www.mdpi.com/2077-0375/10/3/39/s1, Figure S1: BSA static binding capacities at different equilibrium times for both surface modified electrospun PSf-GMA-DEA (7 min UV polymerization time) membrane and mPAN-GMA-DEA (15 min UV polymerization time) membrane. For both membranes, four pieces of membranes were soaked in 4 beakers separately containing 3 mg/mL BSA in 20 mM Tris buffer at pH 7.0. The equilibration process was conducted at room temperature under gentle shaking. Protein binding capacities were measured after 1, 5, 12, 20 h of equilibration from 4 beakers respectively.
