# Supplementary material for: Electrospun Weak Anion-exchange Fibrous Membranes for Protein Purification
Source: Membranes (Basel). 2020 Mar 1;10(3):39. doi: 10.3390/membranes10030039 (PMC7143834; doi:10.3390/membranes10030039)
Supplement: Supplementary file 1 [file membranes-10-00039-s001.pdf]

Supporting Information

Electrospun Weak Anion-exchange Fibrous Membranes for Protein Purification

Shu-Ting Chen [1], S. Ranil Wickramasinghe [1] and Xianghong Qian [2,*]

[1] Department of Chemical Engineering, University of Arkansas, Fayetteville, AR 72701, USA
[2] Department of Biomedical Engineering, University of Arkansas, Fayetteville, AR 72701, USA
* Correspondence: xqian@uark.edu; Tel.: 479-575-8401

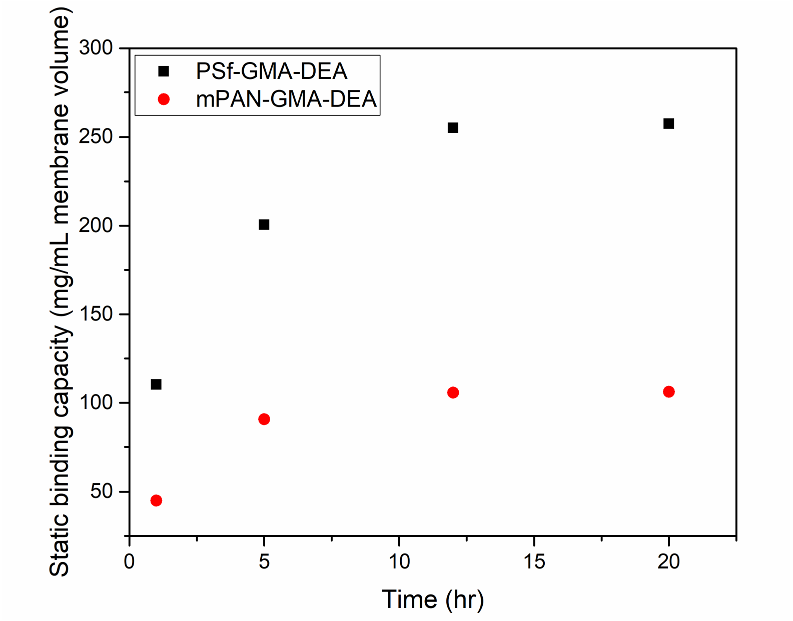

Figure 1. BSA static binding capacities at different equilibrium times for both surface modified electrospun PSf-GMA-DEA (7 min UV polymerization time) membrane and mPAN-GMA-DEA (15 min UV polymerization time) membrane. For both membranes, four pieces of membranes were soaked in 4 beakers separately containing 3 mg/mL BSA in 20 mM Tris buffer at pH 7.0. The equilibration process was conducted at room temperature under gentle shaking. Protein binding capacities were measured after 1, 5, 12, 20 h of equilibration from 4 beakers respectively.
